# Evolution of Ecological Diversity in Biofilms of Pseudomonas aeruginosa by Altered Cyclic Diguanylate Signaling

**DOI:** 10.1128/JB.00048-16

**Published:** 2016-09-09

**Authors:** Kenneth M. Flynn, Gabrielle Dowell, Thomas M. Johnson, Benjamin J. Koestler, Christopher M. Waters, Vaughn S. Cooper

**Affiliations:** a Department of Molecular, Cellular, and Biomedical Sciences, University of New Hampshire, Durham, New Hampshire, USA; b Department of Microbiology and Molecular Genetics, Michigan State University, East Lansing, Michigan, USA; c Department of Microbiology and Molecular Genetics, University of Pittsburgh School of Medicine, Pittsburgh, Pennsylvania, USA; Geisel School of Medicine at Dartmouth

## Abstract

The ecological and evolutionary forces that promote and maintain diversity in biofilms are not well understood. To quantify these forces, three Pseudomonas aeruginosa populations were experimentally evolved from strain PA14 in a daily cycle of attachment, assembly, and dispersal for 600 generations. Each biofilm population evolved diverse colony morphologies and mutator genotypes defective in DNA mismatch repair. This diversity enhanced population fitness and biofilm output, owing partly to rare, early colonizing mutants that enhanced attachment of others. Evolved mutants exhibited various levels of the intracellular signal cyclic-di-GMP, which associated with their timing of adherence. Manipulating cyclic-di-GMP levels within individual mutants revealed a network of interactions in the population that depended on various attachment strategies related to this signal. Diversification in biofilms may therefore arise and be reinforced by initial colonists that enable community assembly.

**IMPORTANCE** How biofilm diversity assembles, evolves, and contributes to community function is largely unknown. This presents a major challenge for understanding evolution during chronic infections and during the growth of all surface-associated microbes. We used experimental evolution to probe these dynamics and found that diversity, partly related to altered cyclic-di-GMP levels, arose and persisted due to the emergence of ecological interdependencies related to attachment patterns. Clonal isolates failed to capture population attributes, which points to the need to account for diversity in infections. More broadly, this study offers an experimental framework for linking phenotypic variation to distinct ecological strategies in biofilms and for studying eco-evolutionary interactions.

## INTRODUCTION

Biofilms, or aggregates of microbial cells attached to surfaces, are the primary life history strategy for bacteria found in most environments (1, 2) and are known for their biodiversity (3–5). The process of forming biofilm produces physical layers and resource gradients that generate new ecological opportunities (6, 7), which can select for greater genetic variation and even augment the robustness of the population (8). One of the most concerning examples involves biofilms associated with chronic lung infections of persons with cystic fibrosis (CF), in which distinct colony types that differ in traits such as antibiotic resistance, motility, quorum sensing, and adherence evolve (9–13), resulting in infections that are nearly impossible to eradicate (14–16).

A growing field of research seeks to identify the ecological forces driving diversification and how interactions among mutants evolve. Short-term experimental microbial evolution (EME) studies focusing on biofilm adaptation have been fruitful (for example, see reference 17) and have yielded phenotypes commonly seen during chronic lung infections such as mucoidy, small-colony variants (SCVs), loss of virulence factor production, and changes in cell surface virulence determinants (18–20). To date, most *in vitro* biofilm models track the evolution of populations over only a few hundred generations, whereas pathogens involved in chronic lung infections lasting decades may experience more than 10,000 generations of evolution and involve the coexistence of many lineages (21). Therefore, longer experiments that begin to mimic the duration of chronic infections are necessary to study the consequences of this diversity for subsequent adaptation and population function. The evolution of biotic interactions among subpopulations may profoundly affect the trajectory of adaptation. For instance, clinical isolates of Pseudomonas aeruginosa commonly vary in motility and biofilm production (22). This diversity might represent selection to occupy distinct niches generated by other mutants or species, as has been shown in simple laboratory models (23–25). Understanding the evolution of biofilm diversity demands that these forms of eco-evolutionary feedback be characterized (26).

We previously developed a simple system enabling long-term evolution of bacterial populations under conditions requiring attachment to a plastic bead suspended in a test tube and then, upon transfer to a new tube, dispersal to a new bead (see Fig. S1 in the supplemental material) (27). In studies with Burkholderia cenocepacia, similar, heritable colony phenotypes evolved in each replicate population that associated with distinct ecological strategies (7), including “wrinkly” SCVs that attach to the plastic surface early and appear to inhabit a common niche (28). This ecological diversity associated with mutations affecting proteins or protein complexes that are predicted to affect concentrations of the secondary messenger molecule, cyclic di-GMP (c-di-GMP) (27). The concentration of c-di-GMP governs transitions between motile, planktonic growth and sessile, biofilm attachment in a wide range of organisms and environments (29–31). Together, these findings suggest that clonal biofilms may diversify by selection for types that differ in motility, attachment timing, and/or preferred binding substrate (a foreign surface or other cells) partly by way of altered c-di-GMP metabolism.

Here, we tested the generality of these findings by experimentally evolving another opportunistic lung pathogen, Pseudomonas aeruginosa, which poses a broader threat within the CF community and for other susceptible patients (32). Prior parallelism among replicate B. cenocepacia populations led us to predict similar parallelism among replicate P. aeruginosa biofilm populations founded by strain PA14 and transferred for 600 generations. In sharp contrast, each replicate biofilm population developed unique and unpredictable morphological diversity, which raised a central question of how this diversity relates to the adaptive trajectories taken by each replicate population. We modeled this diversity by constructing synthetic populations comprised of representative isolates from one population with seven colony types. Four of these colony phenotypes were found to independently contribute to the population's ability to colonize a surface, and one minority type proved especially important for early colonization of the plastic surface and disproportionately affected population fitness. We then characterized additional interactions among mutants by disrupting levels of the signaling molecule c-di-GMP in individual clones and measuring the effects on population composition and productivity. Numerous interactions among mutants became evident, suggesting that observed colony types do in fact reflect distinct ecological roles found within our bead transfer system, such as the timing of colonization, attachment substrate, mode of dispersal, and capacity for planktonic growth. More generally, this study offers a novel approach for deciphering ecological networks within populations or communities.

## MATERIALS AND METHODS

### Bacterial strains and growth conditions.

All strains used are derivatives of Pseudomonas aeruginosa PA14, the founder strain for this EME study (33). P. aeruginosa PA14 and derived isolates were grown overnight from freezer stock in a mixture of 1 ml tryptic soy broth (TSB) and 4 ml M63 medium [base consisting of 15 mM (NH_4_)_2_SO_4_, 22 mM KH_2_PO_4_, and 40 mM K_2_HPO_4_ supplemented with 40 mM galactose, 1 mM MgSO_4_, 25 μM FeCl_2_, and 0.4% (wt/vol) arginine]. Planktonic cultures were grown in 5 ml of M63 medium in 18- by 150-mm test tubes on a roller drum at 30 rpm, while biofilm cultures included a 7-mm polystyrene bead suspended in the test tube. When necessary, the following antibiotic concentrations were used: 10 μg/ml gentamicin and 20 μg/ml nalidixic acid for Escherichia coli and 80 μg/ml gentamicin for P. aeruginosa.

### Experimental evolution.

Three replicate populations were grown at 37°C in the presence of 7-mm polystyrene beads suspended in 5 ml of M63 medium in a test tube. Using a method described previously, populations were selected for reversible surface attachment through daily transfer of a bead to a new test tube in which cells must adhere to a new bead in order to persist (see Fig. S1 in the supplemental material) (7). Periodically, a 1-ml aliquot of the old bead and overnight mixture was frozen at −80°C in 8% dimethyl sulfoxide (DMSO). Three replicate planktonic lines were also maintained as a control through the daily transfer of 50 μl of culture under the same conditions lacking a bead. Ninety days of transfers were conducted in total, such that approximately 600 generations of evolution occurred based on ∼6.6 generations/day (see methods in the supplemental material). Colonized beads were added to sterile 15-ml centrifuge tubes and sonicated in 1.5 ml phosphate-buffered saline (PBS) for 10 s, with a Heat Systems Ultrasonics model W-375 sonicator, with an output control of 5 and a duty cycle of 40% to remove all biofilm cells before plating. Sonication was determined to have no effect on the viability of biofilm-grown cells (*t* = 0.31, df = 8, *P* = 0.76) and allowed for more reproducible assessments of bead growth. Prior studies with this model have shown that sonication removes nearly all cells from the beads, based on scanning electron microscopy (7). However, we cannot rule out that some mutants remain more adherent than others, which would ultimately be maladaptive in this system.

### Phenotypic characterization of distinct population members.

Replication populations that evolved under biofilm or planktonic conditions (hereafter referred to as biofilm populations or planktonic populations) were examined for phenotypic diversity by plating on 1% tryptone agar supplemented with 20 μg/ml Coomassie blue and 40 μg/ml Congo red. This agar allows variation in colony morphology to become apparent due to dye uptake following growth for 24 h at 37°C and an additional 48 h at room temperature. Colony morphology was easily resolved on plates with less than 200 CFU, and patterns and frequencies of morphology types were tested and verified as repeatable from 3 to 5 plates of samples from any given time point at multiple dilutions. Representative mutants from each population at each time point were isolated, confirmed as heritable following 24 h of growth in M63 medium, and scored for phenotypes as described below.

Biofilm production was quantified as described previously (34) after 4, 8, and 24 h, with 8-fold replication, with some adjustments. To determine if biofilms formed by the complete B1 population after 24 h were more productive than the sum of the constituent parts, a predicted 24-h population biofilm value was calculated from the biofilm production of each individual type when grown in isolation, scaled for the frequency observed within the population (Table 1), and then summed. A similar approach was performed to determine if the population was more productive in cellular yield, i.e., CFU/ml. Swarm motility was assayed using 0.5% agar as described previously (35). Five microliters of an approximately 1 × 10^8^ CFU/ml suspension was spot plated into the center of dry plates in triplicate. After 48 h of incubation at 37°C, the plates were photographed and the Feret diameters were calculated using ImageJ. Swimming motility was assayed using 0.3% agar with 3-fold replication and a paired wild-type (WT) PA14 control for each replicate, as described previously (36).

**TABLE 1:**
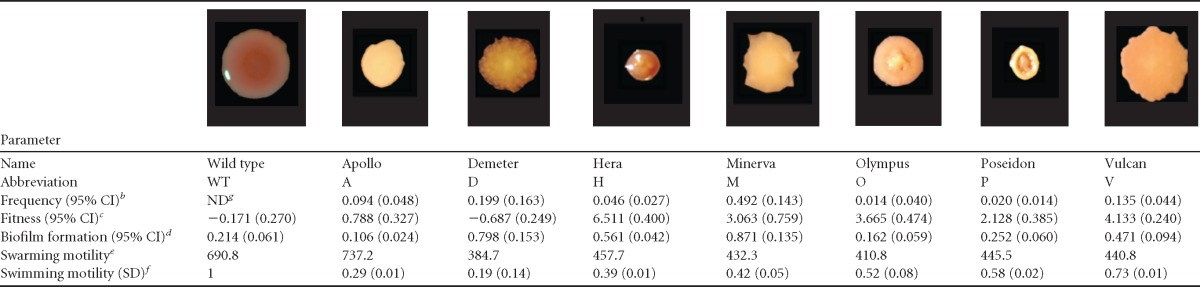
Characteristics of representative clones from the B1 population isolated after 600 generations of biofilm adaptation^*a*^

a Colony images were scaled to their relative sizes on a plate (Fig. 1).

^b^ Identified through repeat plating of diverse mixtures. ND, not detected.

^c^ Selection rate constant measured as the ability to exclude the ancestor of the clone in the selective environment.

^d^ Attachment assayed after 24 h of growth on the walls of 96-well polystyrene plates.

^e^ Based on the Feret diameter of colonies after 48 h at 37°C on minimal growth medium.

^f^ Motility on 0.3% agar relative to that of WT PA14 on the same plate (*n* = 3).

^g^ ND, not detected.

### Biofilm facilitation assay.

To assess whether the attachment of one population member facilitated the attachment of another, one type was allowed to establish a biofilm in wells of a 96-well plate with 100 μl M63 medium for 4 h before the addition of an equivalent quantity of a second type, using independent cultures standardized for optical density. These assays were conducted in microplates with 7-fold replication to enable sufficient replication to resolve pairwise interactions, as the biofilms grown on beads in test tubes were more variable, even among replicates of the ancestor. Biofilms were stained with 1.0% crystal violet (CV) and solubilized with 95% ethanol after 24 h of growth.

### Fitness assays.

Fitness of the evolved clones was determined by competition against a PA14 *attB*::*lacZ* strain (37), as described previously (7), with at least 4-fold replication with minor modifications. All competitors were grown in isolation overnight, harvested from the plastic bead, combined equally in a 50-μl volume, and then added to a tube containing 5 ml of M63 medium with a new bead. Some evolved clones were so successful in competition with the ancestor that starting ratios were skewed to 1:100 to produce countable final ratios. The numbers of CFU/ml of competitors were determined at 0 and 24 h by plating onto 1% tryptone supplemented with X-Gal (5-bromo-4-chloro-3-indolyl-β-d-galactopyranoside). Fitness was calculated as the selection rate, *r*, which is the difference (mutant minus ancestor) in the natural logarithm of realized growth over 24 h. Since less-fit competitors can produce fewer CFU on the bead than were added initially, selection rate is a more appropriate metric than relative fitness (38).

### *In situ* manipulation of c-di-GMP and tracking of population effects.

Populations were reconstructed by mixing members together in the relative frequencies observed in the evolved 600-generation B1 population (Table 1), with one strain harboring the inducible phosphodiesterase (PDE) BifA (pbifA, pMQ80-His-bifA) or the inducible diguanylate cyclase (DGC) SadC (psadC) (39, 40). Plasmids were introduced into PA14 by electroporation (41) or through conjugal transfer mediated by the SM10 λpir strain of Escherichia coli. Expression of *bifA* or *sadC* was induced by adding 0.5% arabinose to the medium. The change in relative abundance was defined as the difference between observed frequencies after 24 h of growth and the frequencies found in the inoculum for 3 to 6 independent replicates. These manipulated communities were then compared to the untransformed constructed community (four biological replicates). Goodness-of-fit G-tests were used to assess the effects of these manipulations on population composition relative to control mixtures.

Methods for genome sequencing, confocal microscopy, and analysis and quantification of c-di-GMP levels are described in methods in the supplemental material.

### Accession number(s).

Genome sequence data are available under NCBI BioProject accession no. PRJNA315478.

## RESULTS

Three biofilm populations (B1, B2, and B3) and three control planktonic populations (P1, P2, and P3) of Pseudomonas aeruginosa PA14 were experimentally evolved for approximately 600 generations using a method described previously (7) that is depicted in Fig. S1 in the supplemental material. Sequential samples of biofilm populations grown on an indicator agar revealed the evolution of distinct and heritable colony phenotypes (Fig. 1), while the WT morphology disappeared. Unexpectedly, these colony morphologies differed among replicate populations and even among longitudinal samples of the same population, despite being reproducible. Although qualitatively similar phenotypes were observed (e.g., SCVs and increased exopolysaccharide [EPS] production as evidenced by Congo red uptake), these phenotypes appeared at different intervals and differed in their abilities to persist. For example, “wrinkly” types observed by 110 generations within B1 and B3 samples were never observed within B2 samples. In contrast, the three replicate planktonic populations (P1, P2, and P3) were more consistent and less phenotypically diverse over time, although small-colony types were found within each planktonic population (see Fig. S2 in the supplemental material). Thus, the initial survey of the phenotypes that evolved from a common clone revealed a surprising variety of colonies for unknown reasons.

**FIG 1:**
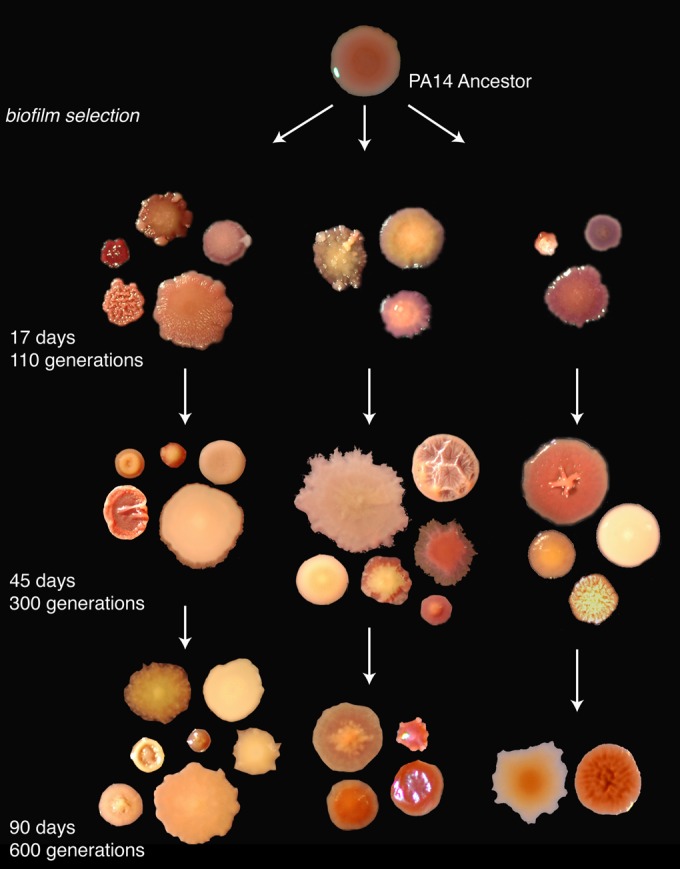
Evolution of morphological diversity within and among three replicate biofilm populations. Aliquots from populations B1 to B3 were isolated after 100, 260, and 600 generations and grown on indicator agar.

### Biofilm populations but not planktonic populations evolved high mutation rates.

One explanation for the unpredictable diversity of colony types is that each replicate biofilm population evolved approximately 50-fold-greater rates of mutation (see Fig. S3 and Table S1 in the supplemental material). We sequenced the genomes of complete population samples and identified likely causative substitutions in the *mutS* (T112P, which evolved independently on different haplotypes in populations B1 and B2) and *mutL* (D467G, population B3) genes, which were fixed by 300 (B1), 170 (B2), and 600 (B3) generations. The genomic dynamics of these populations are beyond the scope of this study and will be reported elsewhere. The *mutS* substitutions occurred at a highly conserved threonine, which when mutated in E. coli limits protein function by reducing affinity for heteroduplex DNA (42). Meanwhile, the planktonic populations retained the wild-type mutation rate, and no mutations expected to influence the mutation rate were found among planktonic isolates (see Fig. S3).

### Distinct colony types represent different ecological roles.

The evolution of increased mutation rates could have led to various colony morphologies unrelated to adaptation. Specifically, the seven distinct colony phenotypes that were evident in the final population sample of population B1 (Fig. 1) may not represent traits that were under selection or that correspond to distinct lineages. We tested whether these seven colony isolates differed from one another in biofilm-related phenotypes that may have been subject to selection (Table 1). The selected clones indeed differed widely in biofilm production, motility, and most importantly in competitive fitness in comparison to the PA14 ancestor (Table 1; see Fig. S4 in the supplemental material). Surprisingly, mutant “D” was less fit than the ancestor in pairwise competition, which is counterintuitive given the strong selective conditions of our experiment. Meanwhile, other mutants achieved high fitness levels that excluded the ancestor to barely detectable levels (Table 1). We then mixed clones at frequencies corresponding to their observed fractions in the mixed community and tested whether they reproduced attributes of the evolved population. The fitness of this constructed community was not statistically different from that of the complete final B1 community (Table 1; complete community, *r* = 0.421; natural community, *r* = 0.327; *t_6_* = 2.213, *P* = 0.075). However, replicate assays of the constructed community tended to be less fit than samples of the complete evolved population, which implies that meaningful diversity may still have been missed.

**FIG 2:**
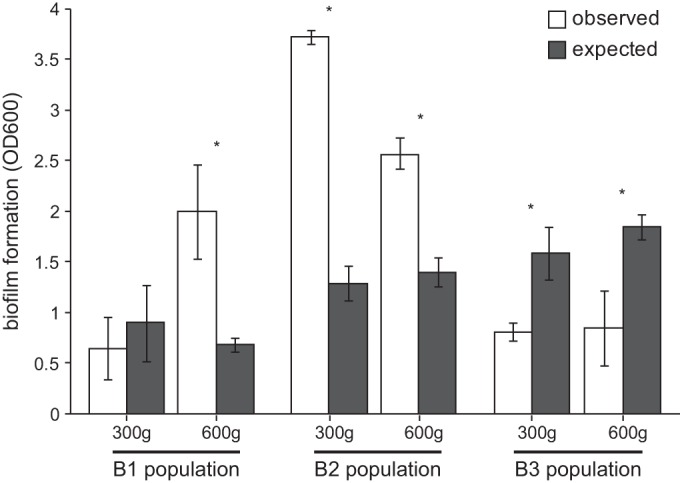
Observed and expected biomasses of evolved populations at two time points. Biofilm formation was measured by crystal violet assays for clones and mixed populations after 300 and 600 generations. The expected effects of mixture are the scaled sum of individual member outputs. Asterisks denote significant differences between observed and expected values within populations, according to *post hoc* Bonferroni comparisons following two-way analysis of variance (ANOVA) (*P* < 0.05). OD600, optical density at 600 nm.

The high variance in competitive fitness among clones versus their common ancestor PA14 indicated that they may interact with one another. We first focused on the sign of these potential interactions, positive or negative. We compared attributes of the B1 clones when grown alone with their properties when mixed together to determine whether their interactions were consistent with negative effects of competition or positive effects of diversity. The number of cells attached to the plastic bead (cellular productivity) of the mixed population was less than the sum of the clonal constituents grown alone, scaled for their mixed frequencies (Table 1; see Table S2 in the supplemental material), suggesting negative effects of competition. However, the biofilm biomass of the final sample of population B1 significantly exceeded the expected outputs of individual clones (Fig. 2; see Table S3). Both early and late samples from population B2 also produced more biofilm than expected from the constituents, but an earlier sample from the B1 population and both samples from population B3 produced less biofilm than expected (Fig. 2; see Table S3), consistent with negative effects of competition.

### Rare variants disproportionately contribute to population fitness.

**FIG 3:**
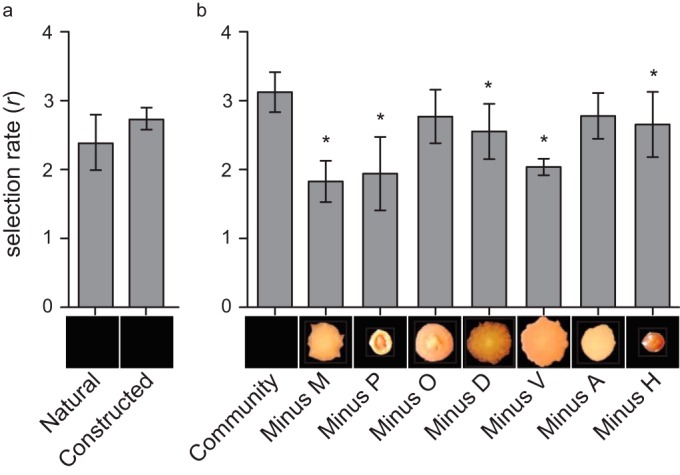
Population fitness requires diversity. (a) Synthetic mixtures of representative clones were used to examine the effect of reduced diversity on fitness for bead colonization of the complete population. The mixtures of all seven morphotypical phenotypes were scaled before inoculation based on their observed relative frequencies reported in Table 1. (b) Incomplete populations, each lacking one of the seven members, were constructed and competed against the ancestor, as was the complete reconstructed population, with at least four replicates. *Post hoc* analysis was performed using Tukey's test with the complete population as the control; asterisks denote a *P* of <0.05. Letter abbreviations refer to a specific B1 isolate: A, Apollo; D, Demeter; H, Hera; M, Minerva; O, Olympus; P, Poseidon; V, Vulcan (Table 1).

To identify potential interactions among the seven members of population B1, we constructed all six-member subsets and quantified how the population responded in the absence of the one morphotype. Populations lacking the M, P, D, V, or H morphotypes lost up to 40% of their fitness advantage over their ancestor (Fig. 3; see Table S4 in the supplemental material), but these fitness losses did not correlate with the biofilm productivity or fitness of the subtracted type (Table 1). For example, removing minority member P or V, which contributed less than 15% to the total cellular yield of the population, reduced fitness by nearly as much as removing the most abundant (∼49%) type, M. Furthermore, type V is clearly the most fit in comparison with the ancestor, whereas type D is actually less fit than the ancestor (Table 1), which suggested that it facilitates rather than excludes its competitor when mixed in equal amounts.

### An early colonizer significantly increases the biofilm output of other community members.

**FIG 4:**
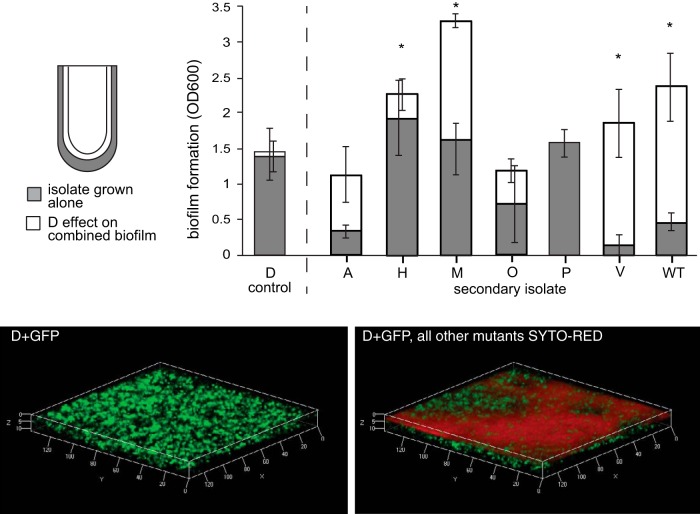
The early colonizing D type facilitates the attachment of other types and enhances biofilm output. (Top) Pairwise biofilm assays were conducted in which the D type was allowed to colonize a polystyrene surface for 4 h before the addition of a secondary isolate. Biofilm formation of individual members growing alone (gray) and the increase in total biofilm following D type colonization (dark gray) are shown. Asterisks signify combinations that are significantly more productive than both the member grown alone and D with more D added to control for greater cell density. (Bottom) Confocal microscopy of the constructed biofilm community inoculated simultaneously with D plus pMQ80-GFP (40) with induced GFP expression. The remainder of the cells were imaged after staining with the red Syto62 dye (Invitrogen). Left, only GFP-expressing cells are shown. Right, combined image of GFP and all red-stained cells attaching to the D type.

One potential mechanism by which different mutants evolving within a biofilm might differentially influence the complete biofilm is in their mechanism or timing of attachment. We surveyed the B1 population members for temporal variation in both biofilm production (see Fig. S5a in the supplemental material) and attachment to a bead (see Fig. S5b). The D and V morphotypes, whose presence enhanced the fitness of the mixed population versus that of the WT (Fig. 3), displayed enhanced attachment early during the growth cycle (see Fig. S5). To test the hypothesis that other members benefit by early D or V type colonization, we assayed the biofilm production of pairwise combinations in which one of these mutants established a biofilm prior to the addition of a secondary isolate. Colonization of the plastic surface by the D type for 4 h led to significantly greater biofilm productivity by the M, H, and V types, as well as the ancestor (Fig. 4). This mixing effect depended on the order of colonization: pairwise mixtures in which M colonized the surface before the addition of the D type produced significantly less biofilm biomass (biofilm formation of D and M mixtures with initial colonization with M type, 2.11 ± 0.34, compared to initial colonization with D type, 2.95 ± 0.30; *t*_14_ = −4.43, *P* = 0.0006). Yet no pairwise combinations with type V differed from additive expectations, suggesting that this ecotype influences population function by some other mechanism (see Fig. S6).

### Altering c-di-GMP metabolism of key mutants reveals evolved ecological interactions.

**FIG 5:**
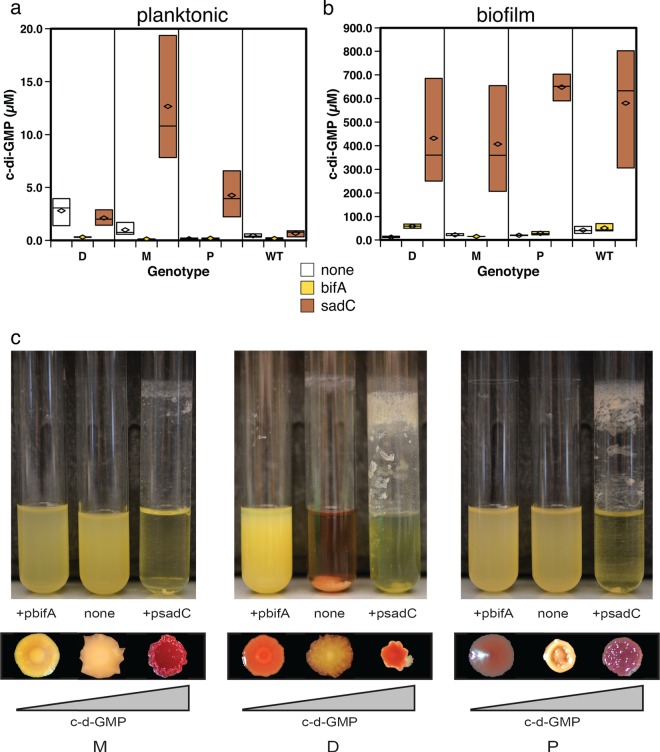
Evolved biofilm mutant physiology can be disrupted by modifying c-di-GMP levels. c-di-GMP was extracted from planktonic culture-grown (a) or biofilm-grown (b) cells of the M, D, and P mutants and the WT. Inducible plasmids were added to each genotype containing *bifA*, a phosphodiesterase gene, or *sadC*, a diguanylate cyclase gene. Box plots depict differences and statistical comparisons between genotypes with induced *bifA* or *sadC* and those without induction (no treatment), as summarized in Table S5 in the supplemental material. (c) Phenotypic effects of altered levels of c-di-GMP as determined from growth patterns in overnight cultures of LB and changes in colony morphology.

Because increased concentrations of c-di-GMP can produce colony variants like those here (Fig. 1) (28, 43, 44), we hypothesized that temporal variation in c-di-GMP levels would correlate with some of the variation in morphology and attachment timing. Cyclic-di-GMP concentrations were quantified from both planktonic and biofilm-grown D, M, P, and WT cells, revealing significant variation in both growth conditions (see Table S5 in the supplemental material). Results of this experiment are depicted in Fig. 5a and b (no treatment, vector controls) and show high basal levels of c-di-GMP in planktonic D cells, which may stimulate their early attachment, and higher levels in WT biofilm-grown cells (Fig. 5; see Table S5).

If variation in c-di-GMP metabolism contributes to the maintenance of population diversity, we reasoned that altering c-di-GMP levels in a single type would disrupt not only its function but perhaps also the balance and function of other types with which it interacts. First, we experimentally manipulated the levels of c-di-GMP *in vivo* for the M, D, and P types as well as the WT and quantified the effects on biofilm phenotypes (Fig. 5c). Plasmids were added to these types with inducible copies of either the phosphodiesterase (PDE) gene *bifA*, which we predicted would reduce intracellular levels of c-di-GMP, or the diguanylate cyclase (DGC) gene *sadC*, which we predicted would increase c-di-GMP levels. As expected, levels were much lower in the planktonic phase than the biofilm phase (Fig. 5a and b). Further, *sadC* expression produced much higher levels than *bifA* expression (see Table S5 in the supplemental material). These experiments also revealed significant differences among mutants: the D mutant retained higher c-di-GMP levels despite *bifA* phosphodiesterase expression during both planktonic and biofilm growth, whereas the M mutant showed a great increase in c-di-GMP levels with *sadC* diguanylate cyclase expression in the planktonic phase. Even more conspicuously, induction of these enzymes in the evolved mutants altered phenotypes associated with c-di-GMP levels in the predicted directions, such as biofilm production, colony morphology, autoaggregation, and pigment secretion (pyoverdine, pyocyanin) of the manipulated clones (Fig. 5c). Expression of *sadC* in the D and P mutants led to hyperbiofilm phenotypes and very little visible planktonic growth (Fig. 5c).

**FIG 6:**
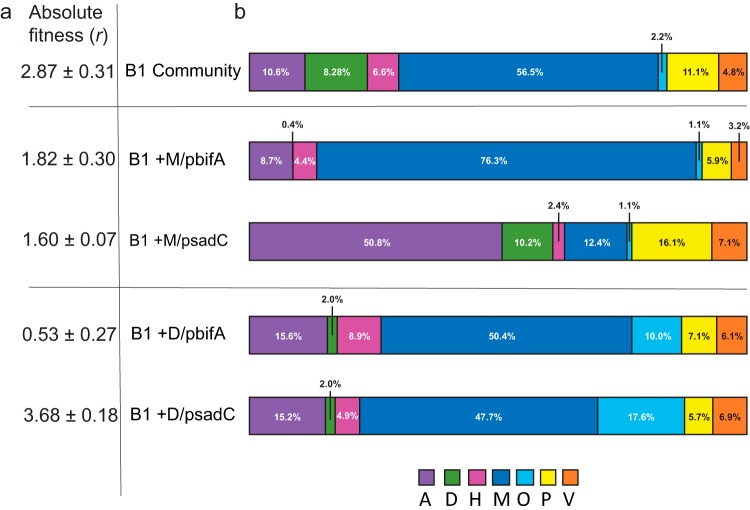
Altering c-di-GMP levels in individual B1 members affects population fitness as a whole. Populations were constructed with certain members expressing an inducible phosphodiesterase gene, *bifA*, which reduces intracellular levels of c-di-GMP, or an inducible diguanylate cyclase gene, *sadC*, which increases c-di-GMP levels. The effects of manipulating a single population member were measured as fitness (a) and composition (b) relative to those of an unmanipulated B1 population. In panel a, the size of the circle represents the magnitude of population fitness versus that of the PA14 ancestor; dotted circles represent 95% confidence intervals. In panel b, bar charts illustrate changing relative frequencies of each member, but not population sizes, which were typically negatively affected by these manipulations.

We then added these mutants with inducible plasmids to constructed communities and evaluated the effects of induction on population fitness and composition, relative to the nontransformed constructed community. Although we cannot rule out a growth effect of adding arabinose as a resource, in nearly all cases this treatment was deleterious for growth. Further, increasing or decreasing c-di-GMP in the targeted genotype also altered the composition of the constructed community (see Table S6 in the supplemental material) and revealed complex interactions (Fig. 6). First, increasing or decreasing c-di-GMP in the D type tended to increase the relative abundance of the O type at its own expense (Fig. 6b; see Table S7). Although the M and H types produced more biofilm following early colonization by D (Fig. 4), the fraction of these types was relatively unaffected when D was manipulated. Remarkably, greater c-di-GMP levels in D increased population fitness (*t_12_* = 3.557, *P* = 0.004), perhaps because its hyperbiofilm phenotype increased adherence by other cells. Second, increased c-di-GMP in the M type reduced population fitness (Fig. 6a), reduced M frequency, and led to a drastic increase in the A type (Fig. 6b). Together, these shifts strongly suggest that the A and O types occupy and compete for similar ecological space as the M and D types, respectively. Lastly, this experiment further illustrates that relatively rare variants can disproportionately affect population function: reducing c-di-GMP levels in the rare high-biofilm producer, P (representing ∼5% of the population) (Table 1), lowered population absolute fitness more than removing the P type from the population entirely (*r =* 1.102 ± 0.255 [95% confidence interval {CI}] versus 2.328 ± 0.685 [95% CI]; *t*_6_ = 5.34, *P* = 0.002). SadC overexpression in the P type produced a corresponding increase in absolute fitness (*r* = 2.48 ± 0.19), whereas *bifA* overexpression in the P type severely compromised fitness (*r* = 2.48 ± 0.26) and enriched for the A and H types at the expense of the M type (see Table S7), which may be evidence of indirect competition between these types. Together, these experiments manipulating a single community member revealed a complex network of interactions among evolved mutants related to their different attachment timing, motility, and proportional abundance. Most clearly, the high biofilm production of the early colonizing D mutant led to multiple interactions that enhanced the biofilm output of the entire community.

## DISCUSSION

Although increased biodiversity appears to be inherent to biofilms, the long-term consequences of this diversity for population function remain understudied. Most studies of biofilm selection have employed models like drip-flow reactors, colony formation, floating pellicles, or flow cells that emphasize persistent attachment to a surface and can only follow adaptation over the short term (5, 22, 24, 45). But chronic infections such as those in the CF lung often involve considerable and persistent variation maintained over years (9–11, 21, 46, 47). These basic and applied problems motivated this long-term study of evolution in P. aeruginosa biofilms involving the daily life cycle of attachment, assembly, and dispersal over 600 generations. We expected to observe diversification in this fluctuating environment, but the level of conspicuous, heritable diversity observed during this experiment both within and among replicate populations in colony morphology (Fig. 1), mutant function (Table 1; Fig. 3 and 4), and population function (Fig. 2) was unexpected.

One explanation is that evolution in structured environments can generate and preserve more diversity than homogenous conditions (48–52). Structured biofilm populations may therefore be expected to increase the likelihood that different beneficial mutants succeed in replicate populations. Previous experiments following evolution in biofilms have not supported this prediction and produced parallel patterns of diversity, perhaps because of the overwhelming strength of selection created by experimental designs involving large population sizes and/or maladapted ancestral genotypes (27, 53, 54). Here, we observe the lack of parallelism that might be expected in heterogeneous environments, yet the diverse mutants are still almost certainly a product of selection. The high fitness of most variants in population B1 relative to that of the ancestor provides strong evidence. We are thus left with the question of how a selection-dominated regime acting on initially clonal populations can generate divergent genetic and ecological outcomes, while other designs have not.

The high biofilm capacity and physiological versatility of the ancestral genotype may provide some insight. Our founder PA14 strain, for example, produces substantial biofilm biomass and is relatively well adapted for growth in our laboratory conditions, which should limit the probability that highly beneficial mutations in the same limiting traits would dominate in replicate populations. This starting condition may not only explain the lack of phenotypic parallelism but also the rise of mutator genotypes in each population. Although the experimental evolution evidently generated strong selection given the resultant large fitness increases, the daily fluctuations of the model may have favored many competing beneficial mutations adapted to distinct phases of the growth cycle (55, 56). The rise of many equivalent contending lineages would increase the probability of mutations affecting DNA repair to hitchhike and, as they rise to high frequency, to become more likely to produce secondary beneficial mutations (57, 58). Thus, an ecologically subdivided population with many beneficial mutations should increase the likelihood of hitchhiking and, in turn, the probability of mutator haplotypes. This explanation is by no means the only one, as the evolution of mutators in P. aeruginosa biofilms has attracted much study (19, 59, 60). However, it does suggest a population genetics explanation that may fit with our practical understanding of this species and even this particular strain, which evidently can grow in many environments and hosts using a broad range of mechanisms (61).

A more straightforward explanation for the divergent evolutionary outcomes within and among these biofilm populations involves “eco-evolutionary feedback” loops (62), or ecological interactions facilitated by the evolution of adapting subpopulations growing in close quarters. In accordance with this theory, this feedback was initiated by the first mutants that arose by chance and prevailed under strong selection, which then altered the environment and selection on other types. Specifically, our findings support a model in which early colonists inhabit the plastic surface and construct new niches for secondary colonists as well as for generalists that balance biofilm and planktonic growth (7, 27, 63). As evidence, four distinct ecological strategies that are required for maximal population fitness and are unable to complement one another (Fig. 3) evolved in the B1 population. Interestingly, each isolate independently contributes to bead colonization, suggesting that this parameter of our selection regime can be subdivided into at least four discrete niches. The distinguishing trait that explained some of this niche subdivision is timing of attachment, such that some variants benefit from attaching to other variants rather than to the plastic surface itself (Fig. 4). Prior studies using this model with a strain of Burkholderia cenocepacia also revealed a similar process of niche differentiation and the evolution of synergistic productivity driven by distinct attachment patterns of evolved mutants (7, 8). Furthermore, this differentiation is associated with the evolution of a population that produces more biofilm than expected from the sum of its parts (Fig. 2). The greater evolved diversity found in these P. aeruginosa populations that started with a better adapted genotype therefore offers the prediction that continued evolution of the B. cenocepacia populations would produce further ecological diversification. Further, in both of the evolved biofilm systems, lacking key early colonists such as the P. aeruginosa D mutant or the B. cenocepacia wrinkly mutant, reduced this productivity and destabilized biofilm output (7, 64), findings that may point the way toward ecologically informed antimicrobial strategies.

Although the precise drivers of these alternative ecological strategies remain under study, one likely regulatory mechanism producing varied attachment timing is altered levels of c-di-GMP. Previous research has identified c-di-GMP levels to influence adaptive variation in biofilms (23–25). Here, we find further evidence that distinct niche specialists may function by different patterns of c-di-GMP signaling. The early colonizing D mutant that facilitated growth of other types in pairwise interactions produced high basal levels of this signal in planktonic culture (Fig. 4), whereas other types showed relatively greater levels in the attached biofilm. This finding suggests that the D type may have evolved to rapidly reattach following dispersal, as required by our daily transfer regime, by mutations affecting basal c-di-GMP levels.

**FIG 7:**
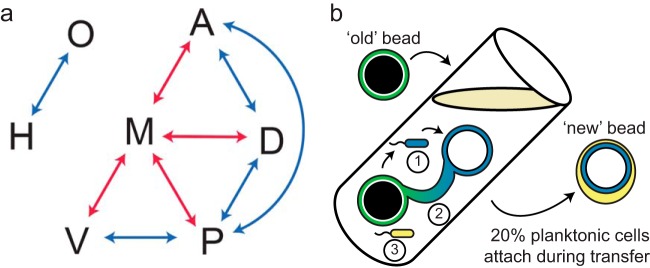
Model of interactions among the seven representative clones from B1 after 600 generations. (a) Experiments that disrupted the function of a single population member (Fig. 6) revealed eight significant linear correlations indicative of potential population interactions (see Table S7 in the supplemental material). Blue arrows signify a positive correlation, while red arrows signify a negative correlation. Correlations were statistically evaluated by a general linear model; a Bonferroni correction was then applied to reduce false positives due to multiple comparisons. (b) Potential mechanisms of bead-to-bead transfer used by PA14 lineages throughout the experiment. We hypothesize that cells can disperse and actively reattach to the new bead (mode 1), transfer via the biofilm mass (mode 2), or survive in the planktonic phase by being transferred along with the beads (mode 3). Each of these mechanisms potentially acts as its own ecological opportunity, enabling persistence of diversity.

We emphasize that selection did not act on c-di-GMP levels *per se* but rather on altered downstream phenotypes affected by this signal, such as the correct timing of growth, attachment, and dispersal (Fig. 5 and 6). Both of the enzymes that we used to modulate c-di-GMP, BifA and SadC, have been shown to pleiotropically regulate biofilm formation (Fig. 5) by modulating EPS production through the *pel* operon and by affecting flagellar function (39, 65). Using these enzymes to alter the physiology of certain mutants revealed at least eight potential pairwise population interactions, importantly involving the other three isolates (A, H, and O types) that were not found to be required for bead colonization (Fig. 7a). We acknowledge that these interactions were defined among clones that were isolated from a diverse community and may not represent the entire population. However, manipulating one genotype led to the rise of one or more genotypes along with the fall of others, which could be explained by these types inhabiting complementary niches. However, the costs of these shifts to population fitness or productivity suggest that the niches of interacting types only partially overlap. For example, inducing BifA in the M type reduced its abundance but increased the abundance of the A type, which implies that A inhabited a niche vacated by M (Fig. 6b). Yet this manipulation reduced population fitness, which might result from the lower biofilm output of the A type (Table 1) and also suggests why removing A from the population altogether did not statistically affect fitness (Fig. 3). Another pairwise interaction between the O and H types appeared to occur independently of the other types when the population was disrupted (Fig. 7). We hypothesize that the O and H types may have adapted to other fitness parameters of our bead selection regime.

PA14 cells may successfully proliferate in our system through a variety of mechanisms and, by extension, within multiple ecological niches. First, cells may disperse to the planktonic phase and actively reattach to the next bead before transfer (Fig. 7b, mode 1). However, the timing of attachment can further subdivide this niche (Fig. 4; see Fig. S5 in the supplemental material). Second, cells may not be required to disperse; bead-associated PA14 biofilms may occasionally grow to connect both beads within a culture. As such, biofilm specialists may potentially transfer from bead to bead without transitioning to the planktonic lifestyle (Fig. 7b, mode 2). Third, planktonic-phase specialists that do not attach may also persist (Fig. 7b, mode 3), since ∼20% of the cells transferred on a bead originate from the planktonic phase (see methods in the supplemental material).

We are still far from a complete model that assigns distinct ecological roles to all seven colony types within the evolved P. aeruginosa population studied here and even further from a general model of P. aeruginosa biofilm ecology. However, understanding long-term adaptation in biofilms must build upon a more intimate grasp of the magnitudes of local interactions and the variety of ecological differences that influence evolution. This study also offers an experimental framework to help microbial ecologists begin to dissect the ecology and population genetic dynamics of biofilms, including the unpredictable mixtures such as those found in chronic lung infections of persons with CF (10). Individual infections tend to be highly unique; large differences in the abundance of certain resident species or isolates with specific phenotypes are not uncommon. Furthermore, these infectious communities are highly dynamic through time, with the majority of variation occurring within rather than between patients. These confusing patterns are remarkably similar to those described here (Fig. 1) and suggest that interactions among types that evolve over long time scales can lead to variable and unpredictable mutant phenotypes. Future studies using reproducible and archivable models of evolution such as ours under conditions that more closely resemble CF sputum (i.e., synthetic CF medium [SCFM]), or under other conditions that restrict growth rates, would provide an important test of this hypothesis. Further, the degree of unpredictability among experimental or infectious biofilms may relate fundamentally to the starting capacity, or fitness, of the founding strain. Somewhat paradoxically, the less adapted the strain, the more deterministic the final ecology might be, because mutants of high benefit exclude others (66, 67). But for many infecting strains of the protean species P. aeruginosa, the populations that evolve from them may be endlessly diverse and hence present continued challenges.

## Supplementary Material

Supplemental material
