# Supplementary material for: Evolution of Ecological Diversity in Biofilms of Pseudomonas aeruginosa by Altered Cyclic Diguanylate Signaling
Source: J Bacteriol. 2016 Sep 9;198(19):2608–18. doi: 10.1128/JB.00048-16 (PMC5019052; doi:10.1128/JB.00048-16)
Supplement: Supplemental material [file supp_198_19_2608__index.html]

Supplemental material 

# Evolution of Ecological Diversity in Biofilms of Pseudomonas aeruginosa by Altered Cyclic Diguanylate Signaling

## Supplemental material

- Supplemental file 1 -

  Supplemental methods

  Fig. S1, method of long-term experimental evolution in biofilms

  Fig. S2, evolution of morphological diversity

  Fig. S3, hypermutation evolved in biofilm populations but not planktonic populations

  Fig. S4, isolates of individual morphotypes tend to be more fit than the community

  Fig. S5, variation in the timing of biofilm formation and attachment

  Fig. S6, the V morphotype does not facilitate the attachment of other types

  Table S1, mutation rates producing ciprofloxacin resistance

  Table S2, comparisons of yields of morphotypes

  Table S3, comparisons between expected and observed levels of biofilm formation

  Table S4, selection rate or fitness of various communities

  Table S5, effects of genotype, growth phase, and treatment

  Table S6, G tests of goodness-of-fit assessing changes in community composition

  Table S7, correlated changes in abundance within diverse mixtures

  PDF, 1.2M
