# Supplementary material for: Evolution of Ecological Diversity in Biofilms of Pseudomonas aeruginosa by Altered Cyclic Diguanylate Signaling
Source: J Bacteriol. 2016 Sep 9;198(19):2608–18. doi: 10.1128/JB.00048-16 (PMC5019052; doi:10.1128/JB.00048-16)
Supplement: Supplemental material [file JB.00048-16_zjb999094061so1.pdf]

Supplementary Methods, Flynn et al., The evolution of ecological diversity in biofilms of *Pseudomonas aeruginosa* by altered cyclic diguanylate signaling.

Growth of *Pseudomonas aeruginosa* PA14 in the bead transfer regime . Biofilm-evolved (B1, B2, B3) and planktonic-evolved (P1, P2, P3) populations were grown in 5 mL of M63 media at 37°C with or without the presence of 7 mm polystyrene beads. Although cells growing in a biofilm are expected to grow more slowly under biofilm conditions, we found no significant difference in the growth rate (μ) when accounting for the carrying capacity of a bead (K) and final population size (N)…

$$\mu = \frac{[\ln(N) - \ln(K)]}{t} \tag{1}$$

$$\text{generations per day} = \frac{\mu}{ln2} \tag{2}$$

Biofilm-evolved cells were found to experience 7.00 ± 0.85 generations per day compared to 6.6 generations per day expected based on the dilution of 50 uL into 5 mL of fresh M63 media performed daily. Although more subtle variation likely exists, we assumed equal rates of growth rate and adaptation under both experimental conditions and report the number of generations of adaptation experienced for populations and isolates based 6.6 generations per day. Consequently, sampling after 17, 25, 44, 66, 75 and 90 days of transfers are assumed to correlate with approximately 110, 170, 300, 440, 500 and 600 generations of adaptation throughout.

We also acknowledge that our method selecting for ‘reversible stickiness’ is inherently complex by design. As such, we typically assume that cells must attach, disperse and reattach to new beads to survive in our system. However, the lack of a rinse step that would exclude purely planktonic cells also means that these cells may not be required to reattach to persist. To determine how many cells may survive bead

transfers without attaching, we rinsed beads colonized overnight by PA14 cells in PBS and subsequently in another overnight culture of PA14 lacZ+ cells before diluting and plating bead-associated cells. Following this treatment, approximately ~20% of the plated cells were lacZ+ (Lac-/Lac+ ratio of 4.03 +/- 0.99 s.d), and hence need not be attached.

Genome sequencing and mutation identification. Samples were sequenced from 27 metagenome samples isolated after 115, 170, 300, 440, 500 and 600 generations from each biofilm-evolved population and 440 and 600 generations from each planktonic-evolved population. This yielded 20.2 giga-base-pairs of sequencing data. Genomic DNA from each population sampled after the final day of experimental evolution was extracted from 1 mL of overnight TSB cultures started from freezer stocks utilizing Qiagen's DNeasy Blood & Tissue Kit. Library construction and sequencing were conducted using the Illumina Nextera DNA Sample Prep Kit and sequenced using the Illumina HiSeq2500 at the Hubbard Center for Genomic Studies (HCGS) at the University of New Hampshire. The BRESEQ software package (1) was used to align reads to the *Pseudomonas aeruginosa* UCBPP-PA14 reference genome (2), and also identify mutations present in each sample. The mutations were manually validated through the examination of BRESEQ's graphical output of aligned reads. Furthermore, we sequenced genomic DNA from our laboratory's ancestral PA14 stock to eliminate pre-existing mutations, and to help eliminate false positives introduced by sequencing and alignment error. These samples enabled us to determine both the likely causes of the mutator phenotypes and the timing of their occurrence within each replicate population. Other results will be reported elsewhere.

Extraction of cyclic-di-GMP. Cyclic-di-GMP was extracted using a nucleotide extraction method described previously followed by liquid chromatography-tandem-mass spectrometry (LC-MS/MS) (3, 4). Briefly, cells were suspended in 250 μL of extraction buffer containing 40:40:20 methanol, acetonitrile, and 0.1 N formic acid. Extraction mixtures were incubated at 20˚C for one hour. After the incubation, 200 μL of the supernatant was transferred to fresh, cold tubes containing 8 μL of 15% ammonium bicarbonate. Samples were dried using a gas manifold and re-suspended in 100 μL of the mobile phase buffer. Due to the instability of c-di-GMP in mass spectrometry mobile phase buffer (10 mM tributylamine and 15 mM acetic acid in 97:3 water/methanol), samples were quantified immediately after processing. Extractions were performed after 17 hours from both planktonic phase and biofilm-associated cells grown under evolution conditions (see above). Cellular yield before each extraction was also determined to allow for standardization of the results following quantification. Values are reported as absolute micromolar concentrations given an estimate of the average volume per cell of $0.83 \mu M^3$.

Confocal microscopy. Constructed communities containing individual members harboring pMQ30 were grown for 24-36 at 37 ˚C with half of a polystyrene cover slip. After growth, biofilm-coated cover slips were carefully removed and placed into a sterile petri dish containing 20 uM SYTO-62 red fluorescent dye (Invitrogen, Carlsbad, CA, USA) for 10 min. After staining of the biofilm, the sample was preserved with a thin layer of 0.1M DABCO. A glass cover slip was supported over the sample with stacked plastic cover slips. Images were taken using a Zeiss 510 META Laser Scanning Microscope (Zeiss, Oberkochen, Germany).

Supplemental References

1. **Deatherage, D. E., and J. E. Barrick.** 2014. Identification of mutations in laboratory-evolved microbes from next-generation sequencing data using breseq. Methods Mol Biol **1151:**165-88.
2. **Lee, D. G., J. M. Urbach, G. Wu, N. T. Liberati, R. L. Feinbaum, S. Miyata, L. T. Diggins, J. He, M. Saucier, E. Deziel, L. Friedman, L. Li, G. Grills, K. Montgomery, R. Kucherlapati, L. G. Rahme, and F. M. Ausubel.** 2006. Genomic analysis reveals that Pseudomonas aeruginosa virulence is combinatorial. Genome Biol **7:**R90.
3. **Massie, J. P., E. L. Reynolds, B. J. Koestler, J. P. Cong, M. Agostoni, and C. M. Waters.** 2012. Quantification of high-specificity cyclic diguanylate signaling. Proc Natl Acad Sci U S A **109:**12746-51.
4. **Newell, P. D., S. Yoshioka, K. L. Hvorecny, R. D. Monds, and G. A. O'Toole.** 2011. Systematic analysis of diguanylate cyclases that promote biofilm formation by Pseudomonas fluorescens Pf0-1. J Bacteriol **193:**4685-4698.

Supplemental Figures S1-6

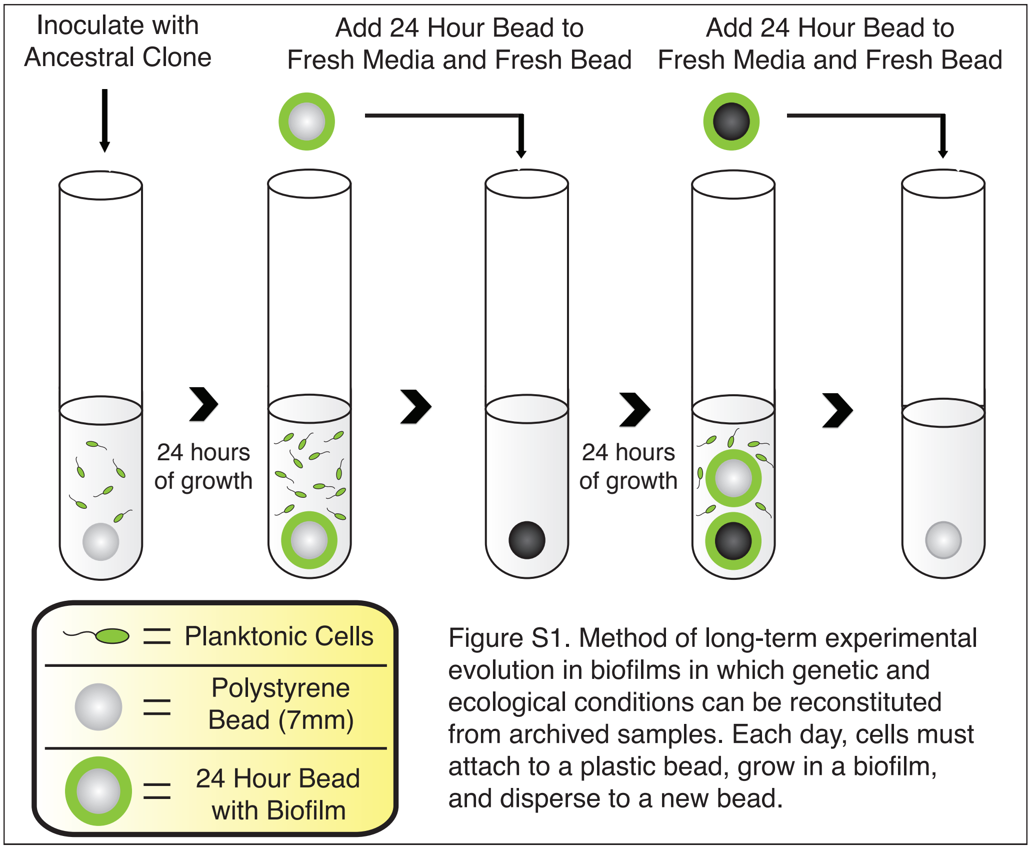

Figure S1. Method of long-term experimental evolution in biofilms in which genetic and ecological conditions can be reconstituted from archived samples. Each day, cells must attach to a plastic bead, grow in a biofilm, and disperse to a new bead.

Figure S1. Method of long-term experimental evolution in biofilms in which genetic and ecological conditions can be reconstituted from archived samples. Each day, cells must attach to a plastic bead, grow in a biofilm, and disperse to a new bead.

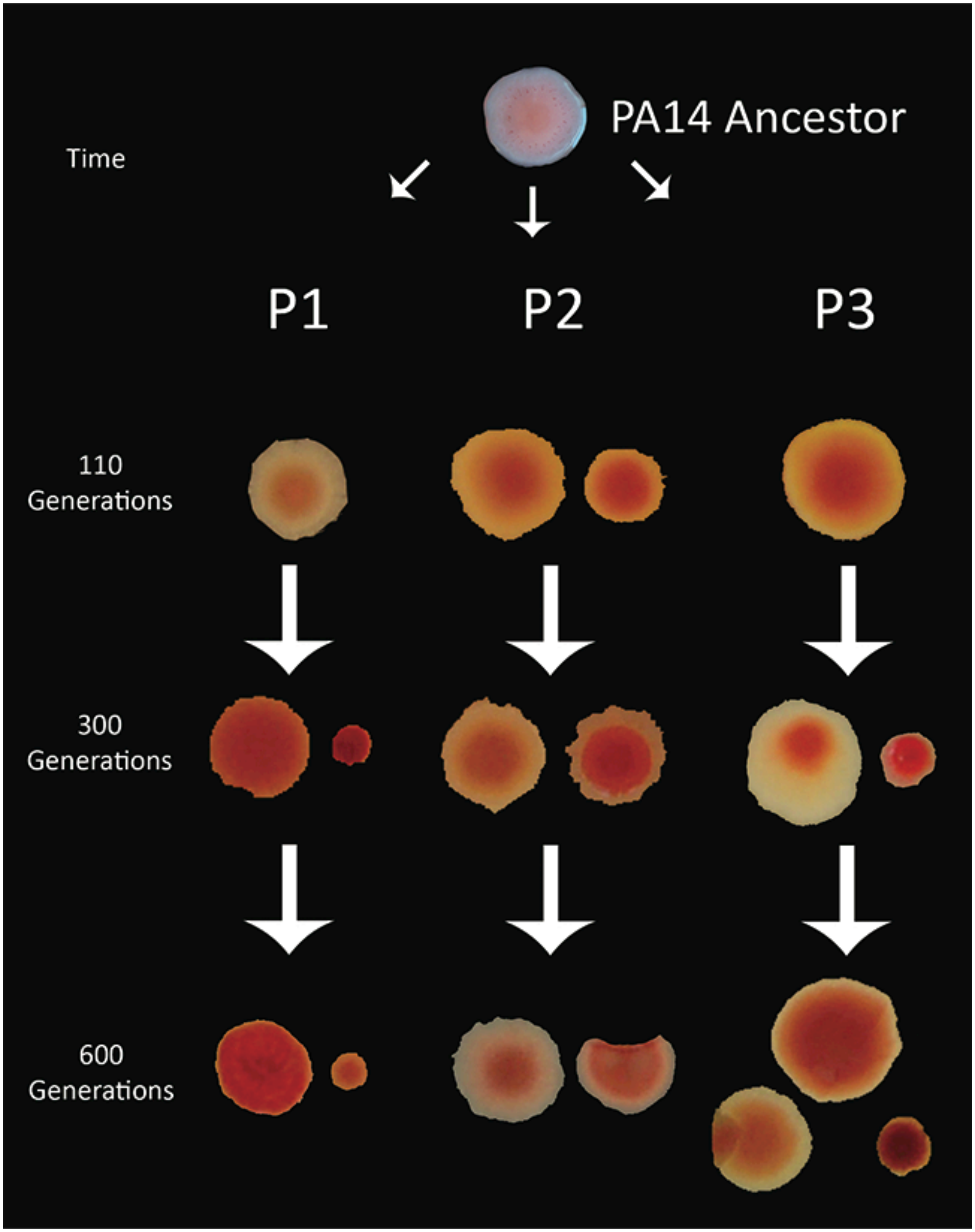

Figure S2. Evolution of morphological diversity among three replicate planktonic populations. Aliquots from different time points were grown on 1% tryptone supplemented with Coomassie blue and Congo red. Number and letter designations (B1-3) refer to the replication population numbers.

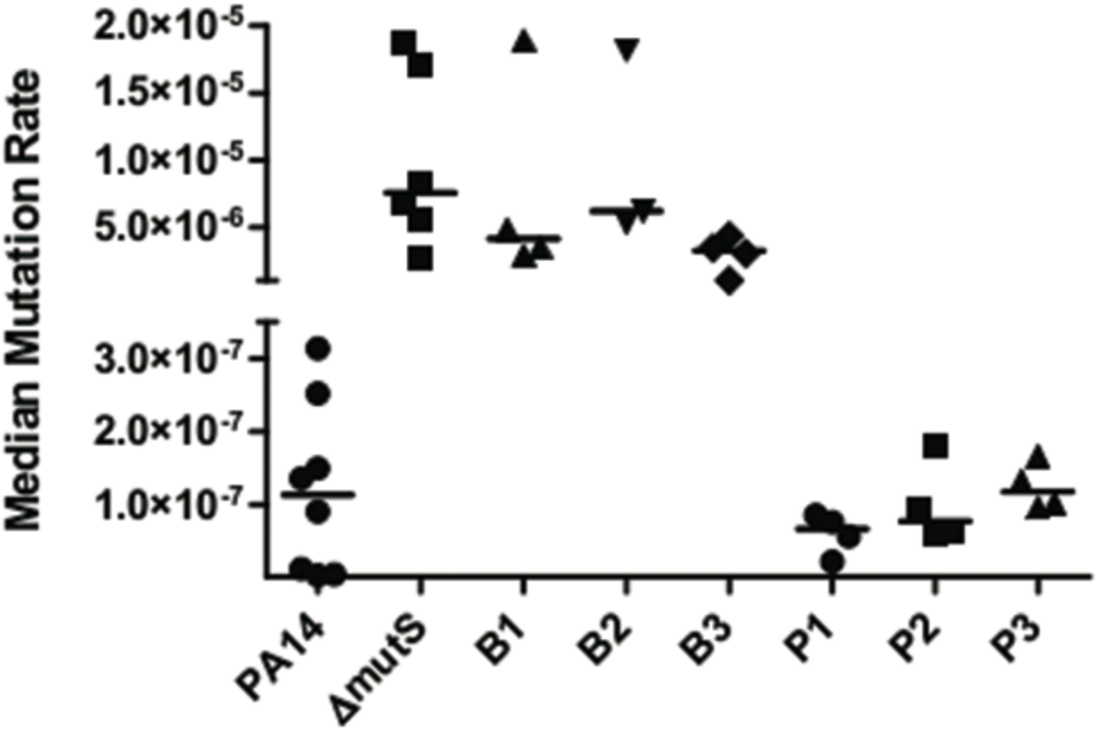

Figure S3. Hypermutation evolved in replicate biofilm populations but not planktonic populations. Fluctuation tests with representative isolates from replicate populations approximated the mutation rate through the spontaneous appearance of mutants resistant to 1 μg/mL ciprofloxacin and 100 μg/mL gentamicin (data not shown). Horizontal bars represent the median rate of mutation.

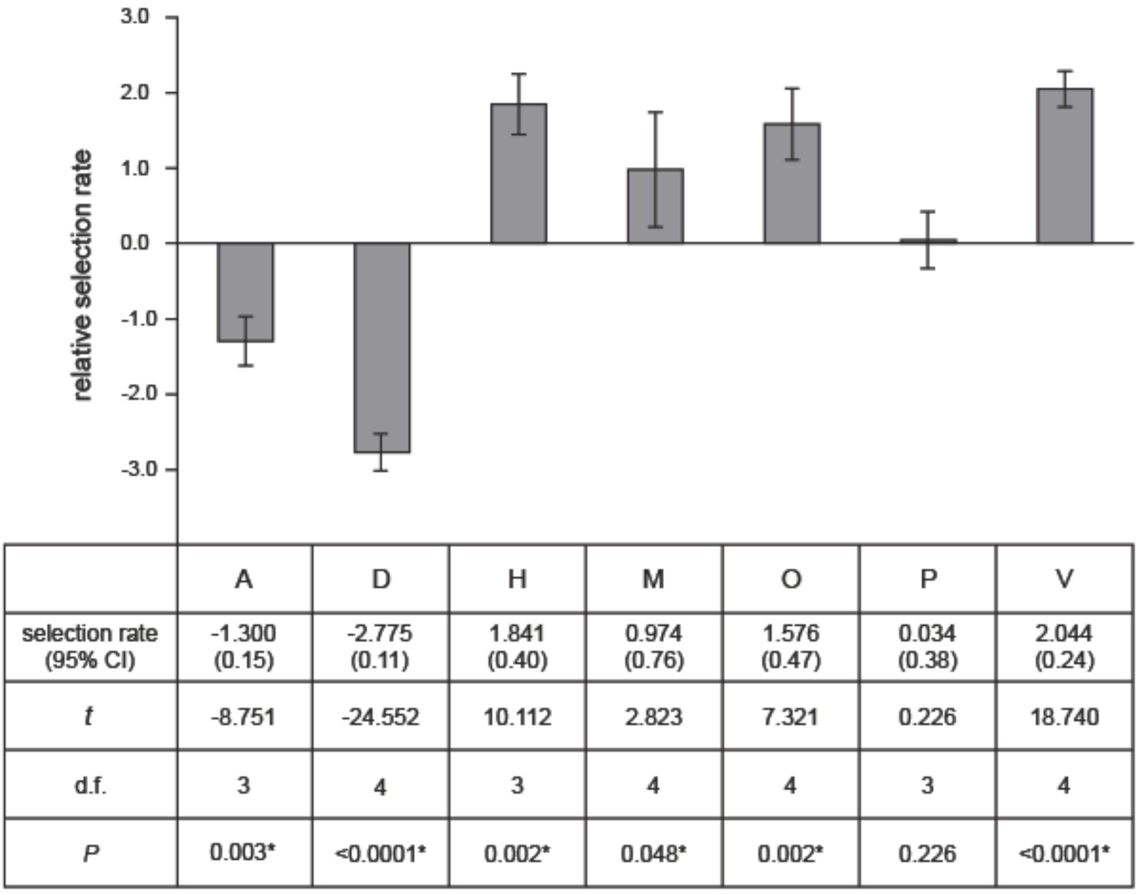

	A	D	H	M	O	P	V
selection rate (95% CI)	-1.300 (0.15)	-2.775 (0.11)	1.841 (0.40)	0.974 (0.76)	1.576 (0.47)	0.034 (0.38)	2.044 (0.24)
t	-8.751	-24.552	10.112	2.823	7.321	0.226	18.740
d.f.	3	4	3	4	4	3	4
P	0.003*	<0.0001*	0.002*	0.048*	0.002*	0.226	<0.0001*

Figure S4. Isolates of individual morphotypes tend to be relatively more fit than the complete community. Individual isolates were competed head-to-head against the PA14 ancestor and standardized relative to the competitive ability of the complete community vs PA14. An experimental mean of one represents equivalency to the complete community. Four out of the seven types present in B1 at the end of the experiment were significantly more fit than the entire community ($P < 0.05$). Included are the t-statistics (t) and degrees of freedom (d.f.) for post-hoc analyses. Numbers in parentheses represent 95% confidence intervals (CI) for relative selection rate values. Letter abbreviations refer to a specific B1 isolate: A = Apollo, D = Demeter, H = Hera, M = Minerva, O = Olympus, P = Poseidon, V = Vulcan (Table 1).

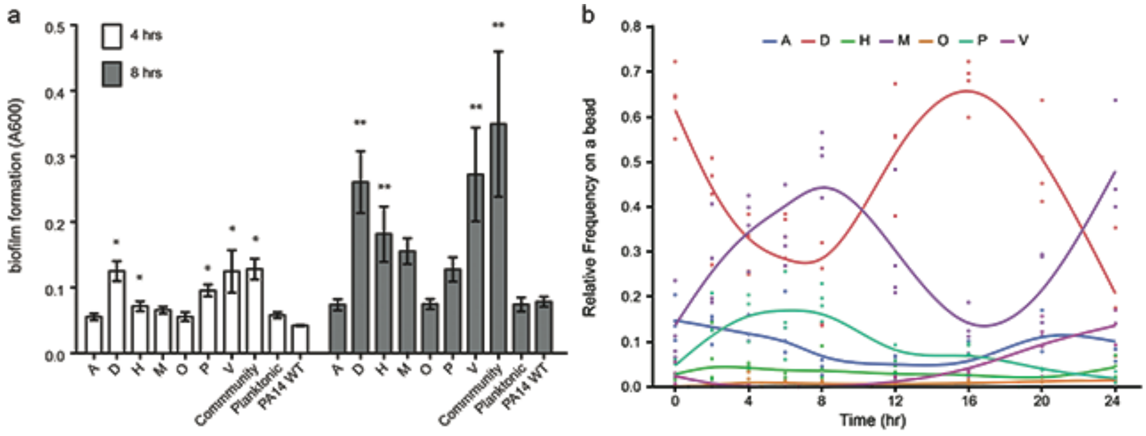

Figure S5. Variation in the timing of biofilm formation and attachment amongst B1 isolates. Isolates from the B1 population after 600 generations of adaptation were examined for variation in the timing of maximum biofilm formation (a) and attachment to a bead (b). a. Biofilm formation after four (white bars) and eight (grey bars) hours of growth. Asterisks denote significance based on post-hoc Tukey tests, $P < 0.05$. b. Replicate cultures were destructively sampled at nine different time points: 0, 2, 4, 6, 8, 12, 16, 20 and 24 hours. Six out of seven morphotypes except H significantly fluctuated in their relative abundance in the community based on CFU/mL on a bead. Asterisks represent significant one-way ANOVAs of abundance through time, $P < 0.05$. Letter abbreviations refer to a specific B1 isolate: A = Apollo, D = Demeter, H = Hera, M = Minerva, O = Olympus, P = Poseidon, V = Vulcan (Table 1).

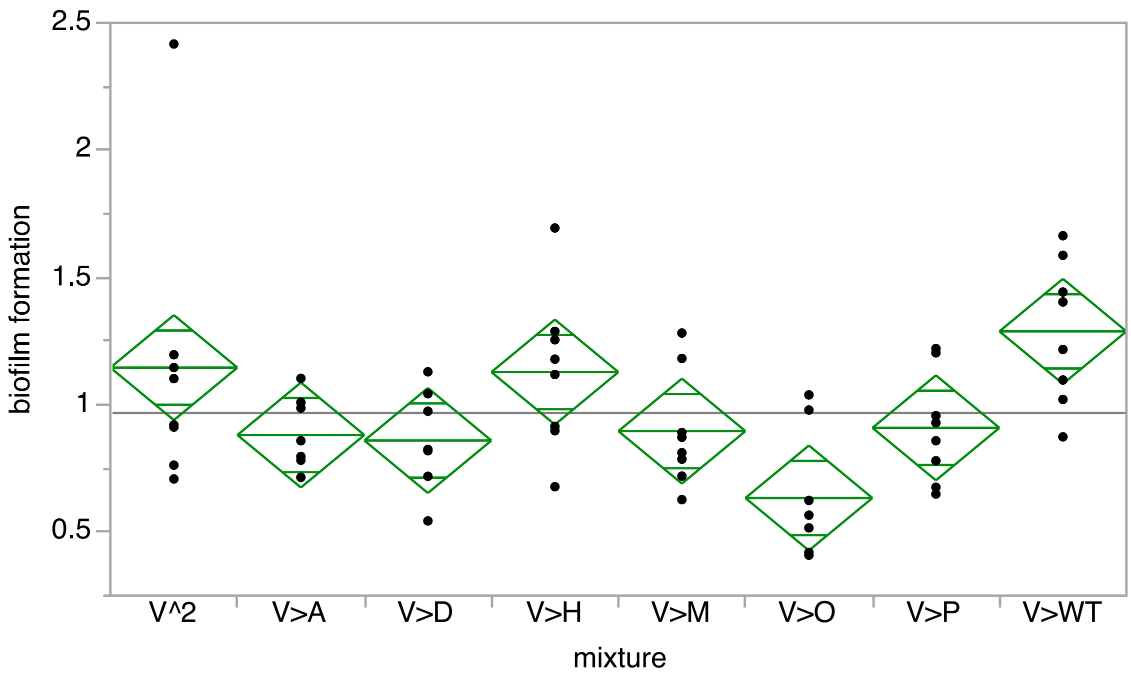

Figure S6. The V morphotype does not facilitate the attachment of other types. Despite a large fitness benefit in monoculture by the V type (Vulcan, Table 1), this type is not able to facilitate the attachment of others to the walls of a 96-well plate unlike the D type (Figure 4). This interaction was assessed by pair-wise biofilm production assays where V was allowed to colonize a surface for four hours before the addition of a secondary isolate.

Table S1. Mutation rates producing ciprofloxacin resistance between 600-generation isolates, a *mutS* deletion strain, and the ancestral PA14 strain. A one-way ANOVA was conducted on results from biological replicates and revealed significant effects of these groupings on log-transformed mutation rates ($F=17.76$, $df=41$, $p<0.0001$). Differences vs WT determined by *post hoc* Dunnett tests are shown here.

**Type**	**Log(Mutation rate)**	**N**	***P vs WT***
**Biofilm-evolved**	-5.803656	19	<0.0001
**Planktonic-evolved**	-7.081625	12	0.808
***mutS* deletion**	-4.996885	6	<0.0001
**PA14 WT**	-7.324980	8	na

Table S2. Comparisons of yield (CFU/mL) of morphotypes grown in monoculture (mono) or community mixture (mix). Comparisons involve independent biological replicates.

**Morphotype***	**Yield (mix)**	**Yield (mono)**	***t***	**d.f.**	***P***
**A**	3.50×10^{6}	3.66×10^{7}	6.539	8	0.0002*
**V**	1.54×10^{6}	1.30×10^{8}	11.23	8	<0.0001*
**P**	3.54×10^{6}	2.61×10^{7}	2.977	8	0.0177*
**M**	2.01×10^{7}	5.42×10^{7}	3.362	8	0.0099*
**H**	2.16×10^{6}	7.34×10^{7}	10.91	7	<0.0001*
**O**	7.40×10^{5}	5.01×10^{7}	4.612	8	0.0017*
**D**	2.78×10^{6}	3.50×10^{7}	4.691	8	0.0016*

* Letter abbreviations refer to a specific B1 isolate: A = Apollo, D = Demeter, H = Hera, M = Minerva, O = Olympus, P = Poseidon, V = Vulcan (Table 1).

Table S3. Comparisons between expected and observed biofilm formation for biofilm-evolved populations after 300 and 600 generations of adaptation. Post-hoc Bonferroni tests were conducted following two-way ANOVA.

**Population**	**Time (gen)**	**Expected biofilm**	**Observed biofilm**	***P***
**B1**	300	0.90412	0.65325	0.3264
**B1**	600	0.69379	2.00795	<0.0001
**B2**	300	1.29909	3.72430	<0.0001
**B2**	600	1.40639	2.56886	<0.0001
**B3**	300	1.59892	0.82009	<0.0001
**B3**	600	1.85427	0.85654	0.0002

Table S4. The selection rate or fitness of various communities constructed from 90-day isolates. Post hoc comparisons following ANOVA to identify homogeneous groupings were conducted using Tukey's test.

**Construct***	**N**	**Selection rate (95% CI)**	**Homogeneous subsets**
**Complete community**	5	3.124 (0.29)	A
**Minus M**	5	1.829 (0.30)	C
**Minus P**	4	1.942 (0.53)	C
**Minus O**	5	2.763 (0.39)	AB
**Minus D**	5	2.553 (0.40)	B
**Minus V**	5	2.039 (0.12)	C
**Minus A**	5	2.779 (0.33)	AB
**Minus H**	5	2.654 (0.47)	B

* Letter abbreviations refer to a specific B1 isolate: A = Apollo, D = Demeter, H = Hera, M = Minerva, O = Olympus, P = Poseidon, V = Vulcan (Table 1).

Table S5. Effects of genotype, growth phase and treatment (induction of phosphodiesterase *bifA*, diguanylate cyclase *sadC*, or no treatment), on c-di-GMP levels. Summary values from one-way ANOVAs.

Growth phase	Treatment	F	df (genotype, error)	p	Post hoc differences
Planktonic	none	8.17	3,8	0.0081	D > WT, M, P
Planktonic	bifA	6.66	3,6	0.0245	D > WT, M, P
Planktonic	sadC	8.40	3,8	0.0075	M > WT,D,P
Biofilm	none	6.50	3,7	0.0196	WT > D,M,P
Biofilm	bifA	8.70	3,7	0.0092	D,WT > M,P
Biofilm	sadC	0.95	3,8	0.4583	n/a
Planktonic		6.18	2,31	0.0055	sadC>bifA,WT
Biofilm		60.45	2,31	<0.0001	sadC>bifA,WT

Table S6. G tests of goodness-of-fit assessing changes in community composition following the disruption of cyclic diguanylate levels in certain mutants.

**Construct**	**df**	**N**	**Pooled G^2**	***P***
**M/pbifA**	6	1142	420.80	9.41×10^{-88}
**M/psadC**	6	614	817.29	2.83×10^{-173}
**D/pbifA**	6	1012	407.77	5.98×10^{-85}
**D/psadC**	6	534	310.53	4.54×10^{-64}
**P/pbifA**	6	275	107.05	8.44×10^{-21}

Table S7. Correlated changes in abundance within diverse mixtures following the disruption of the M and D types by overexpression of *bifA* or *sadC*.

Type	df	Mean Square	*F*	*P*
**A by D**	17	3823.69	4.68	0.046
**A by H**	17	892.89	0.89	0.359
**A by M**	17	11845.60	37.61	1.444×10^{-5}*
**A by O**	17	990.52	1.00	0.333
**A by P**	17	8214.79	15.16	0.001*
**A by V**	17	4114.59	5.15	0.037
**D by H**	17	108.80	0.35	0.565
**D by M**	17	3092.98	24.05	0.0002*
**D by O**	17	42.85	0.13	0.719
**D by P**	17	3192.40	26.08	0.0001*
**D by V**	17	1893.99	9.31	0.008
**H by M**	17	6.70	0.13	0.720
**H by O**	17	510.15	27.03	8.781×10^{-5}*
**H by P**	17	40.72	0.84	0.372
**H by V**	17	3.32	0.07	0.801
**M by O**	17	294.66	0.10	0.762
**M by P**	17	34100.80	34.87	2.217×10^{-5}*
**M by V**	17	24589.50	15.64	0.001*
**O by P**	17	194.29	1.44	0.248
**O by V**	17	69.50	0.49	0.496
**P by V**	17	633.74	13.47	0.002*
